# Self-Reported Knowledge, Correct Knowledge and use of UK Drinking Guidelines Among a Representative Sample of the English Population

**DOI:** 10.1093/alcalc/agx127

**Published:** 2018-01-17

**Authors:** Penny Buykx, Jessica Li, Lucy Gavens, Lucie Hooper, Elena Gomes de Matos, John Holmes

**Affiliations:** 1 ScHARR, University of Sheffield, Regent Court, 30 Regent Street, Sheffield, UK; 2 dosomething.org, 19 W 21st St, New York, NY, USA; 3 Policy Research Centre for Cancer Prevention, Cancer Research, Angel Building, 407 St John Street, London, UK; 4 Epidemiology and Diagnostics, Institut für Therapieforschung, Parzivalstraβe 25, München, Germany

## Abstract

**Aims:**

Promotion of lower risk drinking guidelines is a commonly used public health intervention with various purposes, including communicating alcohol consumption risks, informing drinkers’ decision-making and, potentially, changing behaviour. UK drinking guidelines were revised in 2016. To inform potential promotion of the new guidelines, we aimed to examine public knowledge and use of the previous drinking guidelines, including by population subgroup.

**Methods:**

A demographically representative, cross-sectional online survey of 2100 adults living in England in July 2015 (i.e. two decades after adoption of previous guidelines and prior to introduction of new guidelines). Univariate and multivariate logistic regressions examined associations between demographic variables, alcohol consumption (AUDIT-C), smoking, and knowledge of health conditions and *self-reported knowledge* and use of drinking guidelines. Multinomial logistic regression examined the same set of variables in relation to *accurate knowledge* of drinking guidelines (underestimation, accurate-estimation, overestimation).

**Results:**

In total, 37.8% of drinkers self-reported knowing their own-gender drinking guideline, of whom 66.2% gave an accurate estimate. Compared to accurate estimation, underestimation was associated with male gender, lower education and AUDIT-C score, while overestimation was associated with smoking. Few (20.8%) reported using guidelines to monitor drinking at least sometimes. Drinking guideline use was associated with higher education, overestimating guidelines and lower AUDIT-C. Correctly endorsing a greater number of health conditions as alcohol-related was associated with self-reported knowledge of guidelines, but was not consistently associated with accurate estimation or use to monitor drinking.

**Conclusions:**

Two decades after their introduction, previous UK drinking guidelines were not well known or used by current drinkers. Those who reported using them tended to overestimate recommended daily limits.

**SHORT SUMMARY:**

We examined public knowledge and use of UK drinking guidelines just before new guidelines were released (2016). Despite previous guidelines being in place for two decades, only one in four drinkers accurately estimated these, with even fewer using guidelines to monitor drinking. Approximately 8% of drinkers overestimated maximum daily limits.

## INTRODUCTION

In an effort to curtail the negative health, economic, and social effects associated with alcohol use, governments the world over seek to implement strategies to reduce alcohol consumption. Drinking guidelines are a public health intervention implemented in many countries, including the UK, with the intention of encouraging low to moderate alcohol use and informing the public’s decision-making around alcohol (Heather, 2012). Guidelines typically involve the identification of an amount of alcohol beyond which consumption is seen to pose a significant health risk; although the definition of ‘significant’ is often not explicit. There is variation between countries in how guidelines are set and structured, for example, in the method used for deriving the recommended threshold, the metric used to define a standard drink or unit, whether a daily or weekly threshold is set and whether this differs for men and women (International Center for Alcohol Policies, 2003; Rehm and Patra, 2012; Room and Rehm, 2012; Kalinowski and Humphreys, 2016).

In comparison to other interventions intended to reduce alcohol consumption and related harms, such as increased prices and reduced availability, there is a paucity of evidence regarding the effectiveness of drinking guidelines (Babor *et al.*, 2010). Although a small number of studies have identified increased awareness of guidelines following promotional campaigns (Grøenæk *et al.*, 2001; Livingston, 2012), there are few studies which specifically evaluate the impact of publicizing drinking guidelines on consumption. The evidence of effects on alcohol consumption for education campaigns and health warnings more broadly is mixed (Anderson *et al.*, 2009), with the latter being linked to increased awareness, but not actual behaviour change (Wilkinson and Room, 2009). Given the lack of strong supporting evidence for their effectiveness, it has been argued that drinking guidelines are simply a politically expedient diversion from the implementation of better evidenced, but less popular alternatives (Casswell, 2012). Nonetheless, it may also be argued that in the interests of informed consumer choice, some form of health guidance regarding alcohol use should be available to the general public. It has also been suggested that guidelines ‘may shift public discourse on alcohol’ (Marteau, 2016) such that prevailing attitudes are more supportive of moderation.

The rationale of ‘informed choice’ is reflected by the current UK Alcohol Strategy which identifies drinking guidelines as a potential mechanism by which to ‘ensure that everyone understands the risks around excessive alcohol consumption to help them make the right choices for themselves and their families’(HM Government, 2012, p. 5). As proposed in the Strategy, the UK drinking guidelines have recently undergone review: initially published in January 2016 and formally adopted in September 2016, new guidelines recommend that both men and women not exceed 14 units of alcohol per week (1 unit = 7.9 g/10 ml ethanol) (Department of Health, 2016). This is in contrast to the previous guidelines published in 1995 which recommended not regularly consuming more than 3–4 units a day for men and 2–3 units a day for women (i.e. if, for comparative purposes, the previous guidelines if multiplied across the week imply a recommendation not to exceed 21–28 units for men and 14–21 for women). Additionally, the new drinking guidelines make explicit their health-related purpose by recommending people drink within limits ‘to keep health risks from drinking alcohol to a low level’ and identifying the increased risk of cancer at any level of consumption (Department of Health, 2016).

The report of a monthly omnibus household survey provided data on guidelines knowledge, awareness and use among the general population of Great Britain from 1997 to 2009 in 10 waves (Office for National Statistics, 2010). In 2009, 74% of respondents had heard of drinking daily limits compared to 54% in 1997, with a greater proportion of heavier drinkers having heard of them than lighter drinkers, but little difference was found between men and women. However, lower proportions were able to correctly identify the guidelines: in 2009 only 44% of respondents were aware of the guideline for men and 52% of the guideline for women (compared to 35% and 39%, respectively, in 1997). Again, those with higher levels of self-reported alcohol consumption were more likely to correctly identify the guidelines. Other UK studies using various measures of awareness and/or knowledge also suggest generally poor understanding of drinking guidelines among adults and school and university students (Webster‐Harrison *et al.*, 2002; Gill and May, 2007; de Visser and Birch, 2012).

Given the recent release of new UK drinking guidelines, it is timely to once more consider levels of public knowledge of the 1995 guidelines (House of Commons Science and Technology Committee, 2012), in order to understand the extent to which and by whom these are understood after more than two decades of stability. We therefore aimed to (1) examine the levels of awareness, knowledge, and use of previous drinking guidelines, (2) identify socio-demographic factors associated with these, and (3) explore the extent to which relevant health behaviours (alcohol use and smoking) and health knowledge were associated with knowledge and use of previous guidelines.

## METHODS

### Recruitment and response rate

In July 2015, a cross-sectional online survey was conducted to examine alcohol-related health knowledge, policy attitudes and consumption behaviour. Vision One, an independent market research company was used to recruit a sample of 2100 adults aged 18 and over. E-mail invitations to participate in a survey about ‘health and lifestyle behaviours’ were sent to 11,846 members of the market research company’s online panel, of whom 50% (*n* = 5929) clicked the ‘Start your survey’ link. After screening for quotas based on the population distribution of gender, age, region and education for England, 42% (*n* = 2480) were eligible to proceed. Following exclusion of 380 respondents who provided incomplete or invalid responses, a final sample of 2100 was obtained (84.7% of those who commenced survey). Ethical approval for the survey was granted by the School of Health and Related Research Ethics Committee, University of Sheffield.

### Measures

#### Self-reported guideline knowledge

Knowledge of official drinking guidelines that were in place at the time of the survey was measured via two questions, ‘Do you know how many alcohol units it is recommended that *men* should not exceed in a day?’ and ‘Do you know how many alcohol units it is recommended that *women* should not exceed in a day?’ A new dichotomous (yes/no) variable *self-reported guideline knowledge* was created to reflect whether men and women knew their own gender guidelines.

#### Accuracy of guideline knowledge

Survey respondents who said ‘yes’ to knowing their own gender guideline were then asked to indicate the respective recommended amount onto a sliding scale (from 0 to 10 units, where each point on the scale was half a unit). To assist in responding, a graphic was shown indicating the alcohol unit content of different types of alcohol in a variety of measures (e.g. one pint of beer; one single measure of spirits). Based on responses given on this scale, a new variable was created to indicate *accuracy of guideline knowledge*: men who reported values between 3 and 4 units inclusive and women who reported values between 2 and 3 units inclusive for their own gender guideline were defined as having ‘accurate’ guideline knowledge, in contrast to those who provided an inaccurate estimate (‘underestimate’ = men <3 units/women <2 units and ‘overestimate’ = men >4 units/women >3 units). Those who were coded as ‘no’ for *self-reported guideline knowledge* above were not asked to indicate the recommended number of units and so were coded as ‘no estimate’ for the *accuracy of guideline knowledge* variable.

#### Guideline use

Respondents who reported knowing their own gender guideline (i.e. *self-reported guideline knowledge*) were also asked about the frequency of using it to keep track of their drinking (five-point scale ‘always’ to ‘never’). Responses were recoded into a new *guideline use* variable, dichotomized as ‘yes’ (‘sometimes’, ‘often’ or ‘always’) and ‘no’ (‘rarely’ or ‘never’).

#### Predictor variables

Respondents were also asked questions regarding their demographic characteristics (age in years, gender, highest level of education [seven categories collapsed into ‘no qualifications’, ‘below degree level’ and ‘degree level or above’] and postcode). Postcode data were used to derive 2015 Index of Multiple Deprivation (IMD) quintiles (UK Government, 2015). The IMD is a measure of deprivation calculated for 32, 844 small areas in England based on seven domains: income; employment; health and disability; education, skills and training; crime; barriers to housing and services; and living environment (Department for Communities and Local Government, 2015). Health behaviour items covered smoking and alcohol consumption, the latter measured using the three-item Alcohol Use Disorders Identification Test (AUDIT-C) score (Bush *et al.*, 1998). Respondents were also asked to indicate which of seven health conditions they thought could result from drinking too much alcohol: cancer, heart disease, diabetes, high cholesterol, liver disease, being overweight or obese, and arthritis. The first six of these were previously used in Buykx *et al.* (2015). Arthritis was added in this study to check the discriminant validity of questions but excluded from the derived total sum of health conditions used as a predictor variable.

### Analysis

Descriptive statistics were generated for demographic variables: frequencies for categorical variables and means and standard deviations for continuous variables. Given our focus here on the use of drinking guidelines, we excluded non-drinkers (i.e. those with an AUDIT-C score = 0, *n* = 250) from all analyses, yielding an analytical sample of 1850 drinkers. This sample was used to examine predictors of two outcome measures: *self-reported guideline knowledge* (yes/no) and *accuracy of guideline knowledge* (no estimate, underestimate, accurate estimate, overestimate). A subsample was created comprising respondents who provided an estimate of their own gender drinking guideline (*n* = 699) and this was used to examine predictors of a third outcome measure: *guideline use* (yes/no). Predictors of self-reported guideline knowledge and guideline use were identified via univariate and multivariate logistic regression models while predictors of accurate guideline knowledge were identified via multinomial logistic regression.

Categorical predictor variables were gender, education (no qualifications, below degree, degree or above), IMD quintile (five categories from most deprived to least deprived) and smoker status (daily or occasional smoker vs. past or never smoker). Continuous predictor variables were age in years, AUDIT-C score (range 1–12), and the number of health conditions endorsed as resulting from drinking too much alcohol (range 0–6). *Accuracy of guideline knowledge* (underestimate, accurate estimate, overestimate) was included as an additional variable in predicting *guideline use*. Predictor variables were entered into logistic regression models using the using the default forced entry (i.e. single step) method in SPSS V.22.0 for Windows. As each of the predictor variables were of intuitive relevance to the outcome measures, all were entered into multivariate analyses (Hosmer *et al.*, 2013). Weighting was used in all analyses to adjust for under sampling of respondents without qualifications relative to quotas based on population data for England and Wales from the 2011 Census (Office for National Statistics, 2014).

## RESULTS

### Sample characteristics

Half the sample (50.3%) were male and the average age was 48 years (range 18–80, SD = 16.62); 13% had no educational qualifications, 56% had educational qualifications below university degree level, and 31% were qualified at university degree level or above. The proportions in each population level IMD quintile from most deprived to least deprived were 22.6%, 22.4%, 20.3%, 17.0% and 17.7% (excluding 19 cases for whom postcode data to derive IMD were unavailable). Most respondents were low risk drinkers, the average AUDIT-C score was 4.7 (range 1–12, SD = 2.78), and 33% were daily or occasional smokers. The average number of health conditions endorsed as being related to alcohol consumption was 4.1 (out of a possible 6, range 0–6, SD = 1.62).

### Self-reported guideline knowledge, accuracy of guideline knowledge and guideline use

In total, 699 (37.8%) of respondents self-reported knowing the recommended maximum daily number of alcohol units for their own gender (39.5% of females, 36.2% of males). Of these, 66.2% (*n* = 463) accurately estimated, 13.8% (*n* = 96) underestimated, and 20.1% (*n* = 140) overestimated the number of daily units within their gender’s drinking guideline. Just over half (55.1%, *n* = 385) of those who self-reported knowing their own gender guideline, whether or not they accurately estimated this, reported using the guideline at least sometimes to keep track of their drinking (or 20.8% of all respondents).

Self-reported knowledge, accurate knowledge and use of drinking guidelines by participant characteristics are presented in Tables 1, 2 and 5. At the univariate level, those who were more highly educated, younger, reported more health conditions to be associated with heavy drinking, and had higher AUDIT-C scores were more likely to self-report knowing their own gender drinking guideline (Table 1). With the exception of age, the adjusted odds for all of these predictors remained significant within multivariate regression. Level of education had the highest adjusted odds, with those with a degree or higher level of education being 3.13 times (95% CI [2.13–4.60]) as likely to indicate knowing their own gender guideline compared to those without qualifications. For each additional health condition they endorsed as alcohol-related, respondents were 1.20 (95% CI [1.13–1.28]) times as likely to indicate knowing their own gender guideline and 1.10 (95% CI [1.06–1.14]) times as likely for each additional AUDIT-C point scored. Gender also became significant when controlling for all predictors, females were 1.24 (95% CI [1.02–1.52]) times as likely as males to indicate knowing their own gender drinking guideline. An interaction term between gender and AUDIT-C score was included in a separate regression model (data not shown) to test this association as it was thought the emergence of gender as a significant predictor in the multivariate analysis may have been related to known gender differences in alcohol consumption (with males consuming more on average) (Health and Social Care Information Centre, 2015), however this interaction was not statistically significant.

**Table 1.** Frequency and predictors of self-reported guideline knowledge (*N* = 1850)

Characteristic	*n*^a^	‘Do you know how many alcohol units it is recommended that [own gender] should not exceed in a day?’							
		Yes (*n* = 699)	No (*n* = 1151)	Univariate predictors of ‘yes’ (*n* = 1831)^b^			Multivariate predictors of ‘yes’ (*n* = 1831)^b^		
		%	%	OR	95% CI	*P* value	AOR	95% CI	*P* value
Overall	1850	37.8	62.2						
Gender									
Male	931	36.2	63.8	1.00			1.00		
Female	919	39.5	60.5	1.15	(0.96–1.39)	0.140	**1.24**	**(1.02–1.52)**	**0.036**
Education									
No qualifications	241	19.9	80.1	1.00			1.00		
Below degree	1037	36.7	63.3	**2.34**	**(1.67**–**3.30)**	**<0.001**	**2.08**	**(1.45**–**2.98)**	**<0.001**
Degree or above	573	47.3	52.7	**3.62**	**(2.53**–**5.17)**	**<0.001**	**3.13**	**(2.13**–**4.60)**	**<0.001**
IMD quintile^b^									
5 Most deprived	414	39.4	60.6	0.94	(0.70–1.27)	0.698	1.10	(0.80–1.52)	0.553
4	409	34.7	65.3	0.78	(0.58–1.05)	0.100	0.87	(0.64–1.19)	0.393
3	372	37.3	62.7	0.87	(0.64–1.18)	0.359	0.95	(0.69–1.31)	0.754
2	311	37.3	62.7	0.87	(0.63–1.20)	0.398	0.93	(0.67–1.29)	0.655
1 Least deprived	324	40.7	59.3	1.00			1.00		
Smoker status									
No	1239	37.1	62.9	1.00			1.00		
Yes	611	39.3	60.7	1.09	(0.90–1.34)	0.375	0.96	(0.77–1.20)	0.700
		Mean (SD)	Mean (SD)						
Age*		46.8 (16.9)	48.5 (16.4)	**0.99**	**(0.99**–**1.00)**	**0.031**	1.00	(1.00–1.01)	0.757
No. of health conditions linked to heavy drinking*^c^		4.4 (1.5)	3.9 (1.7)	**1.23**	**(1.16**–**1.31)**	**<0.001**	**1.20**	**(1.13**–**1.28)**	**<0.001**
AUDIT C score*		5.2 (2.8)	4.4 (2.7)	**1.10**	**(1.06**–**1.14)**	**<0.001**	**1.10**	**(1.06**–**1.14)**	**<0.001**

OR = odds-ratios; AOR = adjusted odds-ratios; 95% CI = 95% confidence interval; SD = standard deviation. *OR/AOR per year/unit of increase.

^a^Cell count totals may vary compared to overall sample size due to rounding.

^b^Missing IMD cases (*n* = 19) are not presented here and were excluded from logistic regressions.

^c^Total number of reported conditions linked to drinking too much out of the following six conditions: cancer, heart disease, diabetes, high cholesterol, liver disease, being overweight or obese.

Table results shown in bold are significant (*P* < 0.05).

**Table 2.** Accuracy of guideline knowledge: frequencies (*N* = 1850)

Characteristic	*n*^a^	Estimated number of alcohol units it is recommended that [own gender] should not exceed in a day			
		No estimate (*n* = 1151)	Underestimate (*n* = 96)^d^	Accurate estimate (*n* = 463)^d^	Overestimate (*n* = 140)^d^
		%	%	%	%
Overall	1850	62.2	5.2	25.0	7.6
Gender					
Male	931	63.8	5.9	22.3	7.9
Female	919	60.5	4.5	27.7	7.3
Education					
No qualifications	241	80.1	4.6	11.6	3.7
Below degree	1037	63.3	6.1	24.3	6.4
Degree or above	572	52.8	4.0	31.8	11.4
IMD quintile^b^					
5 Most deprived	414	60.6	4.8	24.4	10.1
4	410	65.1	4.4	21.2	9.3
3	374	62.6	7.5	25.1	4.8
2	312	62.5	5.4	25.0	7.1
1 Least deprived	323	59.4	4.3	29.7	6.5
Smoker status					
No	1239	63.0	5.3	25.6	6.1
Yes	611	60.7	4.9	23.7	10.6
		Mean (SD)	Mean (SD)	Mean (SD)	Mean (SD)
Age		48.5 (16.4)	48.2 (17.3)	47.6 (16.9)	43.4 (16.5)
No. of health conditions linked to heavy drinking^c^		3.9 (1.7)	4.3 (1.5)	4.4 (1.4)	4.4 (1.7)
AUDIT C score		4.4 (2.7)	4.2 (2.7)	5.1 (2.6)	5.9 (3.0)

SD = standard deviation.

^a^Cell count totals vary compared to overall sample size due to rounding.

^b^Missing IMD cases (*n* = 19) are not presented here.

^c^Total number of reported conditions linked to drinking too much out of the following six conditions: cancer, heart disease, diabetes, high cholesterol, liver disease, being overweight or obese.

^d^Underestimate = men <3 units, women <2 units; accurate estimate = men 3–4 units, women 2–3 units; overestimate = men >4 units, women >3 units.

When accuracy of drinking guideline estimation was considered by each predictor variable separately, males were significantly more likely to provide no estimate or an underestimate than an accurate estimate compare to females, as were those with no educational qualifications compared to those with a degree, and those with lower compared to higher AUDIT C scores (Table 3). Identifying fewer health conditions as being related to heavy drinking was significantly associated with providing no drinking guideline estimate compared to an accurate estimate. Overestimation of drinking guidelines compared to accurate estimation was associated with being in the two most deprived IMD quintiles (compared to least), being a smoker, and having a higher AUDIT C score.

**Table 3.** Accuracy of guideline knowledge: univariate analysis of predictors of accuracy in estimating own-gender drinking guidelines (*N* = 1850)

Characteristic	No estimate vs accurate estimate^c^			Underestimate vs accurate estimate^c^			Overestimate vs accurate estimate^c^		
	OR	95% CI	*P* value	OR	95% CI	*P* value	OR	95% CI	*P* value
Gender									
Male	1.00			1.00			1.00		
Female	**0.76**	**(0.62–0.95)**	**0.015**	**0.62**	**(0.40–0.96)**	**0.033**	0.74	(0.51–1.08)	0.116
Education									
No qualifications	1.00			1.00			1.00		
Below degree	**0.38**	**(0.25–0.58)**	**<0.001**	0.66	(0.31–1.41)	0.286	0.84	(0.38–1.88)	0.673
Degree or above	**0.24**	**(0.16–0.38)**	**<0.001**	**0.34**	**(0.15–0.77)**	**0.01**	1.15	(0.51–2.57)	0.738
IMD quintile^a^									
5 Most deprived	1.24	(0.89–1.74)	0.203	1.33	(0.63–2.79)	0.452	**1.85**	**(1.03–3.35)**	**0.041**
4	**1.54**	**(1.09–2.18)**	**0.014**	1.40	(0.65–2.99)	0.388	**1.97**	**(1.08–3.60)**	**0.028**
3	1.25	(0.89–1.76)	0.199	**2.03**	**(1.01–4.11)**	**0.048**	0.85	(0.43–1.70)	0.643
2	1.26	(0.88–1.81)	0.207	1.48	(0.68–3.20)	0.319	1.29	(0.66–2.51)	0.451
1 Least deprived	1.00			1.00			1.00		
Smoker status									
No	1.00			1.00			1.00		
Yes	1.04	(0.83–1.31)	0.727	0.98	(0.61–1.58)	0.935	**1.87**	**(1.27–2.75)**	**0.001**
Age*	1.00	(1.00–1.01)	0.288	1.00	(0.99–1.02)	0.746	**0.99**	**(0.97–1.00)**	**0.010**
No. of health conditions linked to heavy drinking*^b^	**0.80**	**(0.74–0.86)**	**<0.001**	0.95	(0.82–1.10)	0.514	0.96	(0.85–1.10)	0.575
Audit score*	**0.91**	**(0.88–0.95)**	**<0.001**	**0.88**	**(0.81–0.96)**	**0.003**	**1.09**	**(1.02–1.16)**	**0.012**

OR = odds-ratios; 95% CI = 95% confidence interval; SD = standard deviation. *OR per year/unit of increase.

^a^Missing IMD cases (*n* = 19) were excluded from the logistic regression. Analyses were conducted only on valid cases where the outcome variable and all predictor variables are non-missing.

^b^Total number of reported conditions linked to drinking too much out of the following six conditions: cancer, heart disease, diabetes, high cholesterol, liver disease, being overweight or obese.

^c^Underestimate = men <3 units, women <2 units; accurate estimate = men 3–4 units, women 2–3 units; overestimate = men >4 units, women >3 units.

Table results shown in bold are significant (*P* < 0.05).

Multinomial logistic regression analysis including all predictor variables showed that compared to those who accurately estimated their own gender drinking guideline, those who *did not provide an estimate* were more likely to be male, to have no educational qualifications, to report fewer health conditions as being linked to heavy drinking and to have a lower AUDIT C score (Table 4). Predictors of *underestimation* of own gender drinking guidelines (compared to accurate estimation) were being male, having a below degree level qualification (compared to no qualification) and having a lower AUDIT C score. The only significant predictor of *overestimation* compared to accurate estimation was being a current smoker.

**Table 4.** Accuracy of guideline knowledge: multinomial analysis of predictors of accuracy in estimating own-gender drinking guidelines (*N* = 1831^a^)

Characteristic	No estimate vs accurate estimate^c^			Underestimate vs accurate estimate^c^			Overestimated vs accurate estimate^c^		
	AOR	95% CI	*P* value	AOR	95% CI	*P* value	AOR	95% CI	*P* value
Gender									
Male	1.00			1.00			1.00		
Female	**0.70**	**(0.55–0.88)**	**0.002**	**0.51**	**(0.32–0.82)**	**0.005**	0.79	(0.53–1.18)	0.256
Education									
No qualifications	1.00			1.00			1.00		
Below degree	0.43	(0.28–0.66)	<0.001	0.72	(0.33–1.60)	0.425	0.79	(0.34–1.82)	0.580
Degree or above	0.28	(0.18–0.45)	<0.001	0.39	(0.16–0.95)	0.037	1.10	(0.47–2.58)	0.830
IMD quintile									
5 Most deprived	1.01	(0.70–1.45)	0.968	1.24	(0.57–2.68)	0.589	1.50	(0.81–2.77)	0.200
4	1.33	(0.93–1.90)	0.122	1.30	(0.60–2.81)	0.509	1.75	(0.94–3.23)	0.076
3	1.12	(0.79–1.60)	0.518	1.90	(0.93–3.88)	0.079	0.79	(0.39–1.59)	0.505
2	1.17	(0.81–1.69)	0.411	1.34	(0.63–2.98)	0.431	1.28	(0.65–2.50)	0.479
1 Least deprived	1.00			1.00			1.00		
Smoker status									
No	1.00			1.00			1.00		
Yes	1.16	(0.89–1.50)	0.274	1.06	(0.63–1.78)	0.837	**1.54**	**(1.01–2.33)**	**0.045**
Age*	1.00	(0.99–1.00)	0.299	1.00	(0.98–1.01)	0.544	0.99	(0.98–1.00)	0.068
No. of health conditions linked to heavy drinking*^b^	**0.82**	**(0.77–0.89)**	**<0.001**	0.98	(0.84–1.14)	0.757	0.96	(0.85–1.09)	0.558
Audit score*	**0.90**	**(0.86–0.94)**	**<0.001**	**0.86**	**(0.79–0.94)**	**0.001**	1.05	(0.98–1.13)	0.194

AOR = adjusted odds-ratios; 95% CI = 95% confidence interval; SD = standard deviation. *AOR per year/unit of increase.

^a^Missing IMD cases (*n* = 19) were excluded from the logistic regression. Analyses were conducted only on valid cases where the outcome variable and all predictor variables are non-missing.

^b^Total number of reported conditions linked to drinking too much out of the following six conditions: cancer, heart disease, diabetes, high cholesterol, liver disease, being overweight or obese.

^c^Underestimate = men <3 units, women <2 units; accurate estimate = men 3–4 units, women 2–3 units; overestimate = men >4 units, women >3 units.

Table results shown in bold are significant (*P* < 0.05).

Self-reported use of drinking guidelines was associated with a degree or higher level of education, overestimation of the guidelines and higher AUDIT-C scores at the univariate level (Table 5) and these associations remained significant in the multivariate analysis (Table 5). Those with at least a degree level education compared to those with no qualifications were 2.07 times (95% CI [1.06–4.06]) more likely to report using their own-gender guideline to track their own drinking. Those who overestimated their own-gender guideline daily limit were 2.51 (95% CI [1.64–3.86]) times more likely to report using their guideline compared to those who had accurate knowledge. Those with a higher AUDIT-C score were significantly less likely to report using their own-gender guidelines. Unlike self-reported knowledge, the number of health conditions reported as alcohol related was not a predictor of actually using the guidelines to monitor drinking.

**Table 5.** Frequency and predictors of self-reported guideline use to keep track of drinking at least sometimes (*N* = 699)

Characteristic	Use [own gender] drinking guidelines to keep track of drinking at least sometimes										
	Total sample (*n* = 1850)		Of those with self-reported guideline knowledge			Univariate predictors of guideline use			Multivariate predictors of guideline use		
		Yes		Yes (*n* = 385)	No (*n* = 314)						
	*n*^a^	%	*n*^a^	%	%	OR	95% CI	*P* value	AOR	95% CI	*P* value
Overall	1850	20.8	699	55.1	44.9						
Gender											
Male	931	19.2	336	53.3	46.7	1.00			1.00		
Female	919	22.4	363	56.7	43.3	1.15	(0.85–1.55)	0.365	0.98	(0.71–1.37)	0.919
Education											
No qualifications	241	7.9	47	40.4	59.6	1.00			1.00		
Below degree	1037	20.0	381	54.3	45.7	1.74	(0.94–3.20)	0.077	1.78	(0.94–3.39)	0.078
Degree or above	572	27.6	270	58.5	41.5	**2.05**	**(1.10–3.83)**	**0.024**	**2.07**	**(1.06–4.06)**	**0.034**
IMD quintile^b^											
5 Most deprived	414	23.4	163	59.5	40.5	1.22	(0.76–1.93)	0.412	1.23	(0.75–2.03)	0.416
4	410	17.6	142	50.7	49.3	0.84	(0.52–1.36)	0.480	0.81	(0.49–1.34)	0.415
3	374	21.1	139	56.8	43.2	1.08	(0.67–1.74)	0.764	1.15	(0.70–1.89)	0.591
2	312	18.9	116	50.9	49.1	0.85	(0.52–1.41)	0.531	0.89	(0.53–1.49)	0.652
1 Least deprived	323	22.3	131	55.0	45.0	1.00			1.00		
Smoker status											
No	1239	19.9	459	53.6	46.4	1.00			1.00		
Yes	611	22.7	240	57.9	42.1	1.19	(0.87–1.64)	0.273	1.27	(0.89–1.82)	0.186
Correct knowledge of guidelines^c^											
Underestimated			97	46.4	53.6	0.80	(0.51–1.24)	0.311	0.73	(0.46–1.15)	0.175
Correct			463	52.1	47.9	1.00			1.00		
Overestimated			141	70.9	29.1	**2.27**	**(1.51–3.42)**	**<0.001**	**2.51**	**(1.64–3.86)**	**<0.001**
				Mean (SD)	Mean (SD)						
Age*				45.9 (17.2)	47.9 (16.5)	0.99	(0.98–1.00)	0.131	1.00	(0.99–1.01)	0.714
No. health cond’s linked drinking*^d^				4.4 (1.6)	4.4 (1.4)	0.98	(0.89–1.09)	0.723	1.01	(0.91–1.12)	0.895
AUDIT C score*				4.9 (2.5)	5.5 (3.0)	**0.93**	**(0.88–0.98)**	**0.009**	**0.88**	**(0.83–0.94)**	**<0.001**

OR = odds-ratios; AOR = adjusted odds-ratios; 95% CI = 95% confidence interval; SD = standard deviation. *OR/AOR per unit/year increase.

^a^Cell count totals vary due to rounding.

^b^Missing IMD cases (*n* = 8) excluded from regression analyses.

^c^Underestimated (males <3 units; females <2 units); correct (males 3–4 units; females 2-3 units); overestimated (males >4.5 units, females >3 units).

^d^Total number of reported conditions linked to drinking too much out of: cancer, heart disease, diabetes, high cholesterol, liver disease, being overweight or obese.

Table results shown in bold are significant (*P* < 0.05).

## DISCUSSION

In the context of the publication of new UK lower risk weekly drinking guidelines, our findings regarding knowledge and use of the previous daily guidelines are relevant to those with an interest in or responsibility for their promotion. Despite the previous UK drinking guidelines being in place for 20 years (House of Commons Science and Technology Committee, 2012) (including promotion through various media including product labelling, TV, radio and print public information campaigns, health professionals contacts with patients and point-of-sale advertising), among people who drink (i.e. AUDIT C score >0), only about a quarter of people in our study were able to provide a correct estimate of how many units it was recommended their gender should not exceed in a day. This finding indicates lower levels of awareness than in 2009 (Office for National Statistics, 2010), suggesting previous efforts to raise awareness of recommended drinking limits have not had lasting effect.

We further identified characteristics significantly associated with those who think they know, actually do know, and use their own-gender drinking guidelines. Being female, better educated, able to identify more alcohol-related health conditions and consuming more alcohol (as measured by AUDIT C) were all significantly and positively associated with not only claiming to know the drinking guidelines, but also in providing an accurate correct gender-specific estimate of the recommended daily maximum number of units (as they were at the time this study was conducted) compared to not providing an estimate. The same variables, excepting identification of alcohol-related health conditions, were also associated with providing a correct guideline estimate compared to an underestimate. However, when those who claimed to know the guidelines were then asked whether or not they used them to self-monitor their own drinking, only having the highest level of education (i.e. degree or above) remained a positive predictor of guideline use, while unsurprisingly, those who drank at higher levels were significantly less likely to report using guidelines to keep track of their drinking. Unexpectedly, those who overestimated their own-gender guideline were 2.5 times more likely to report using it to keep track of their drinking than those who provided a correct estimate, suggesting there may be a risk that some people drink more than recommended, but on the erroneous assumption that they are consuming within the guideline thresholds. It is of interest to understand whether this issue is now even more pronounced for males since the announcement of new guidelines, which lowered the recommended limits for men. There did not appear to be any inequality in knowledge or use of guidelines according to social gradient as measured by IMD, age or smoking status.

Our findings have relevance for the targeting of promotional activities around the new guidelines. The variable most strongly positively associated with knowledge and use of guidelines was level of education, which suggests that in promoting the new drinking guidelines, efforts should be made to ensure people with lower educational qualifications are an explicitly identified target audience. The new guidelines may be simpler to remember than the old ones because there are no gender-specific recommendations, a single number and less ambiguity around the meaning of ‘regular’. However, the fact that the recommended limits for men have changed to a greater degree than for women, when considered in conjunction with our finding that men were less likely than women to self-report knowing or to actually know the previous guidelines suggests that decisions regarding appropriate promotional avenues for the new guidelines should still take account of gender to ensure reach. That greater awareness of alcohol-related health conditions predicted self-reported knowledge of guidelines but not their use is also an important finding for those designing promotional strategies for the newly released guidelines. Consistent with research regarding the effectiveness of health promotion campaigns (Babor *et al.*, 2010; Dixon *et al.*, 2015), this result suggests that merely understanding the potentially negative health consequences of drinking may be insufficient to change behaviour, whether through reducing consumption or, as measured here, through adoption of guidelines as a self-monitoring strategy. Further, qualitative research suggested that some drinkers perceive daily drinking guidelines to be irrelevant to their drinking patterns (which may involve less frequent but more heavy consumption) and to lack credibility, particularly when their foundation or purpose is unclear to the individual (Lovatt *et al.*, 2015). It is not clear whether the new guidance will be perceived as more relevant in recommending a weekly rather than daily maximum, albeit within reduced overall limits. Alternatively, as might be suggested by our finding that those who overestimated the previous guidelines were more likely than those who accurately estimated them to report using guidelines to keep track of their drinking, the new guidelines might be perceived as less relevant given their more conservative recommendations.

Our study used an online survey methodology and those who opt to participate in such panels may differ from the general population in important ways that we are unable to detect. However, our robust method of quota sampling with additional adjustment through weighting to improve representation related to education has ensured a nationally representative sample with respect to core socio-demographic variables. Levels of survey uptake and completion were also positive for this type of study. A commonly faced alcohol research problem is the potential for inaccuracies in self-reported alcohol use, whether by social desirability or incorrect understanding of what is meant by an alcohol ‘unit’ (Kerr and Stockwell, 2013). To address this potential limitation, which may have also affected estimations of own-gender guidelines, participants were at relevant points in the survey provided with a diagram showing how many alcohol units are in drinks of different size and strength to aid them in their responses.

The low levels of knowledge of previous guidelines found in this study suggest plenty of scope to increase public awareness. Teaching drinkers with unit-marked glasses was found to increase their understanding of guidelines but not alter their consumption (de Visser *et al.*, 2017). While we are aware of some dissemination channels for the previous UK drinking guidelines, such as health promotion materials, we do not have any quantifiable information regarding their active promotion over time. Current research by some of our team aims to document regional and national level actions to promote the new UK guidelines over time. Future monitoring of guideline awareness may be able to utilize this information to better understand the population level effect of active promotion.

## CONCLUSION

Twenty years after their introduction in 1995, only a minority of people in England could accurately estimate the UK drinking guidelines, and even fewer used them for the purpose of monitoring their own alcohol consumption.
